# Late Pregnancy Vitamin D Deficiency is Associated with Doubled Odds of Birth Asphyxia and Emergency Caesarean Section: A Prospective Cohort Study

**DOI:** 10.1007/s10995-020-02999-z

**Published:** 2020-08-25

**Authors:** Hanna Augustin, Sinead Mulcahy, Inez Schoenmakers, Maria Bullarbo, Anna Glantz, Anna Winkvist, Linnea Bärebring

**Affiliations:** 1 The Department of Internal Medicine and Clinical Nutrition, Sahlgrenska Academy, University of Gothenburg, Box 459, 40530 Gothenburg, Sweden; 2 Dublin Institute of Technology, Trinity College Dublin, Dublin, Ireland; 3 Nutrition and Bone Health Group, MRC Human Nutrition Research, Cambridge, UK; 4 The Department of Medicine, Faculty of Medicine and Health Sciences, University of East Anglia, Norwich, UK; 5 Södra Älvsborg Hospital, Borås, Sweden; 6 The Department of Obstetrics and Gynecology, Sahlgrenska Academy, University of Gothenburg, Gothenburg, Sweden; 7 Department of Antenatal Care, Primary Care, Gothenburg, Sweden

**Keywords:** 25-hydroxyvitamin D, Caesarean section, Fetal distress, Birth asphyxia, Apgar score

## Abstract

**Objectives:**

The aim of this prospective cohort study was to investigate the associations between maternal vitamin D status in late pregnancy and emergency caesarean section (EMCS) and birth asphyxia, in a population based sample of women in Sweden.

**Methods:**

Pregnant women were recruited at the antenatal care in Sweden and 1832 women were included after exclusion of miscarriages, terminated pregnancies and missing data on vitamin D status. Mode of delivery was retrieved from medical records. EMCS was defined as caesarean section after onset of labour. Birth asphyxia was defined as either 5 min Apgar score < 7 or arterial umbilical cord pH < 7.1. Serum was sampled in the third trimester of pregnancy (T3) and 25-hydroxyvitamin D (25OHD) was analysed by liquid chromatography tandem mass spectrometry. Vitamin D deficiency was defined as 25OHD < 30 nmol/L, and associations were studied using logistic regression analysis and expressed as adjusted odds ratios (AOR).

**Results:**

In total, 141 (7.7%) women had an EMCS and 58 (3.2%) children were born with birth asphyxia. Vitamin D deficiency was only associated with higher odds of EMCS in women without epidural anaesthesia (AOR = 2.01, p = 0.044). Vitamin D deficiency was also associated with higher odds of birth asphyxia (AOR = 2.22, p = 0.044).

**Conclusions for Practice:**

In this Swedish prospective population-based cohort study, vitamin D deficiency in late pregnancy was associated with doubled odds of birth asphyxia and with EMCS in deliveries not aided by epidural anaesthesia. Prevention of vitamin D deficiency among pregnant women may reduce the incidence of EMCS and birth asphyxia. The mechanism behind the findings require further investigation.

## Significance

### What is already known on this subject?

Poor maternal vitamin D status is suggested as a risk factor of delivery by caesarean section and birth asphyxia. However, studies are few and the data are contradictory.

### What this study adds

In this population-based cohort study, maternal vitamin D deficiency doubled the odds of delivering a baby with birth asphyxia and of delivery by emergency caesarean section in women without epidural anaesthesia. The interaction between vitamin D deficiency and epidural anaesthesia might explain disparate results in previous studies, and should be considered in future studies.

## Introduction

Vitamin D status has received increasing attention for its role in healthy pregnancy. We have previously shown that poor maternal vitamin D status, measured as 25-hydroxyvitamin D (25OHD) in serum, is associated with increased risk of preeclampsia (Barebring et al. 2016a, b), small for gestational age, preterm delivery and low birth weight (Barebring et al. 2018). Poor maternal vitamin D status has also been linked to an increased risk of delivery by caesarean section (C-section). Low maternal 25OHD concentration has been linked to increased risk of primary C-section (Merewood et al. 2009), C-section due to prolonged labour (Scholl et al. 2012) or fetal distress and birth asphyxia (Lindqvist et al. 2016).

Delivery by C-section is associated with increased maternal and fetal risks (Karlstrom et al. 2013). The World Health Organization has set an international recommended rate for C-sections at 10–15% (World Health Organization, 1985, 2015). Globally, almost 19% of all births occur by C-section (Betran et al. 2016) and 18% of births in Sweden during 2014 (SCB 2015), including both elective and emergency C-section (EMCS). Risk factors for EMCS are prolonged labour, fetal distress, fetal malpresentation, multifetal delivery, macrosomia (Caughey et al. 2014), high maternal age, high body mass index (BMI) and nulliparity (Mhaske et al. 2015).

The Apgar score taken after delivery assesses the condition of the new-born, and a low score is a sign of reduced vitality and is a risk factor for future health related complications. A 5 min Apgar score < 7 occurs in 0.5% of all new-born infants in Sweden (Thornberg et al. 1995), and is more common among women with an immigrant background (Milsom et al. 2002). The most common reason for low Apgar score in the absence of fetal malformations is birth asphyxia (Hogan et al. 2007), resulting from an impaired blood gas exchange between pregnant woman and foetus. A low pH of the umbilical cord blood is therefore indicative of birth asphyxia (Georgieva et al. 2013).

Some studies have shown an association between poor maternal vitamin D status and delivery by C-section (Merewood et al. 2009; Scholl et al. 2012), whereas others have not (Zhou et al. 2014; Gernand et al. 2015). A small Swedish nested case control study found an association between lower maternal vitamin D status in early pregnancy and EMCS due to fetal distress, and birth asphyxia (Lindqvist et al. 2016). Poor vitamin D status is associated with impaired muscle strength (Kalliokoski et al. 2013), and pelvic floor disorders (Badalian et al. 2010). Also, it could potentially cause rickets-related malformation of the pelvis, which historically has been a reason for obstructed labour (Loudon 1986). These causes could potentially explain why vitamin D deficiency would increase the risk of EMCS and birth asphyxia, but the mechanisms are unknown.

In sum, few data exist on the associations between maternal vitamin D status and complications during delivery and the results are contradictory. The aim was to study the associations between maternal vitamin D status in pregnancy and EMCS and birth asphyxia.

## Methods

### Recruitment

In total, 2125 women were recruited to the Swedish GraviD cohort study when registering for antenatal care in early pregnancy during autumn 2013 or spring 2014. The only exclusion criterion was a pregnancy exceeding 16 gestational weeks at inclusion. In these analyses, women with aborted pregnancy, miscarriage or missing data on vitamin D status were excluded. The study design has previously been described in detail (Barebring et al. 2016a, b). This study was conducted according to the Declaration of Helsinki and all procedures were approved by the Regional Ethics Committee in Gothenburg, Sweden. Written informed consent was obtained from all participants.

### Data Collection

In the third trimester (T3), from gestational week 31 onwards, women had blood drawn for 25OHD analysis, using liquid chromatography tandem mass spectrometry (LC-MS/MS) (Medicinck servive för klinisk kemi, 2014). Vitamin D deficiency was defined as 25OHD serum concentration < 30 nmol/L. After delivery, medical charts from antenatal and obstetrics care were retrieved. Birth asphyxia was defined by arterial umbilical cord pH and Apgar score, as recorded in the medical chart. Arterial umbilical cord pH < 7.1 or 5 min Apgar score 0–6 was considered low, and presence of either was defined as birth asphyxia. EMCS was defined as delivery by C-section after the onset of labour, as recorded in the medical chart. EMCS was divided by fetal, maternal or other indications. Fetal indications included fetal distress, asphyxia or hypoxia. Maternal indications included cephalopelvic disproportion, failure to progress or failed induction. Other indications included placental abruption, placenta praevia, uterine rupture, cord prolapse, fetal malposition or malpresentation.

### Statistical Analysis

The proportions with EMCS and birth asphyxia among women with and without vitamin D deficiency were compared using Pearson chi-square tests. Associations between vitamin D status (25OHD < 30 nmol/l or ≥ 30 nmol/l) and EMCS or birth asphyxia were assessed by logistic regression analysis. All regression analyses were adjusted for maternal age, BMI (obese; yes/no), height, season at blood sampling (Nov-April or May-Oct) and epidural anaesthesia at delivery. Analyses of EMCS were also adjusted for previous history of childbirth (three categories; nulliparous, previous vaginal delivery, previous C-section), while analyses of birth asphyxia were adjusted for nulliparity (yes, no). Adjusted OR (AOR) are presented. Significance was accepted at p < 0.05. Interaction terms between epidural anaesthesia and vitamin D deficiency were explored for both EMCS and birth asphyxia, where p < 0.2 for the interaction term was considered significant. In case of missing 25OHD concentration in third trimester, vitamin D status from the first trimester (available for most women) was used to impute 25OHD, based on the seasonal variation seen in women sampled in the same initial month. These imputed values (N = 150) were only used in a sensitivity analysis, to see that the results were not biased due to drop out. For 13 women, obstetric medical records were missing and considered lost to follow up. Computer software IBM SPSS Statistics version 22.0 was used for all statistical analyses.

The GraviD cohort’s sample size was originally based on power calculations showing that 2000 women were needed to attain 85% power to detect a doubled incidence of gestational hypertension among vitamin D deficient women. Power calculation using a one-sided log-odds ratio test was based upon log-odds estimations and their asymptomatic variance, combined with normal approximations. As the Swedish incidences of gestational hypertension and EMCS are similar at around 8%, the original power calculation was deemed valid also for the outcome EMCS, assuming a doubled incidence (14% vs 7%) of EMCS among vitamin D deficient women.

## Results

In total, 1832 pregnant women were included in the current analyses. In early pregnancy, mean age of the women was 31 years and mean BMI was 24.5 kg/m^2^ (Table 1).

**Table 1:** Characteristics of the 1832 Swedish pregnant women from the GraviD cohort

	Mean	Standard deviation
Age (years) T1	31.3	4.8
BMI (kg/m^2^) T1	24.5	4.2
Maternal height (cm)	166.8	6.4
Gestational age at blood sampling (days)	234.0	12.9
25OHD (nmol/L) T3	74.7	34.4

	N (%)
Birth history	
No previous delivery/nulliparous	742 (40.5)
Previous vaginal delivery	947 (51.7)
Previous delivery by cesarean	143 (7.8)
BMI ≥ 30 kg/m^2^ at T1	183 (10.0)
Tobacco use at T1	83 (4.5)
Single at T1	35 (1.9)
Employment at T1	
Full-time employment	979 (54.2)
Part-time employment	404 (22.4)
On parental leave	94 (5.2)
Unemployed	329 (18.2)

*T1* first trimester, *BMI* body mass index, *T3* third trimester, *25OHD* 25-hydroxyvitamin D

The total rate of C-section was 13.7% and EMCS was 7.7% (N = 141) (Table 2). EMCS was less common among women who previously had given birth vaginally (2.9%), compared to women with previous C-Section (25.9%) and nulliparous women (10.4%) (p < 0.001). Most EMCS were performed on a maternal indication (3.2%), followed by fetal indications (2.7%) and other indications (1.7%). Birth asphyxia was present in 3.2% (N = 58) of the infants (Table 2) and 11 infants (0.6%) were born with a 5 min Apgar score < 7. A total of 50 infants (2.7%) were born with arterial umbilical cord pH < 7.1.

**Table 2:** Rates of vitamin D deficiency, delivery by cesarean section and birth asphyxia in the GraviD cohort

	All		25OHD < 30 nmol/L		25OHD ≥ 30 nmol/L		
	%	N	%	N	%	N	P^a^
Elective caesarean section	4.9	90	3.8	6	5.3	84	0.445
Emergency caesarean section	7.7	141	9.2	15	7.5	126	0.450
Apgar score 5 min < 7	0.6	11	1.9	3	0.5	8	0.029
Arterial umbilical cord pH < 7.1	2.7	50	5.9	7	3.5	43	0.184
Birth asphyxia^b^	3.2	58	5.7	9	3.0	49	0.063
Epidural anaesthesia at delivery	25.3	464	19.1	31	26.0	433	0.057

*T3* third trimester, *25OHD* 25-hydroxyvitamin D, *pH* power of hydrogen

^a^Derived from Pearson’s chi square tests

^b^Either arterial umbilical cord pH < 7.1 or Apgar at 5 min < 7

Women with vitamin D deficiency had a significantly higher proportion of infants born with low Apgar score (p = 0.029) and a trend toward significantly higher proportion of birth asphyxia (p = 0.063) (Table 2). Among women with vitamin D deficiency, 19% used epidural anaesthesia during delivery, compared to 26% among non-deficient women (p = 0.057) (Table 2).

Administration of epidural anaesthesia modified the association (p = 0.112) between vitamin D deficiency and EMCS. In multivariable logistic regression analyses, vitamin D deficiency was only related to higher odds of EMCS among women who had not received epidural anaesthesia (Table 3). Vitamin D deficiency was not associated with EMCS performed on fetal (AOR = 1.28, p = 0.621) or other (AOR = 1.79, p = 0.266) indications, and epidural anaesthesia did not modify these results. However, epidural anaesthesia modified the association between vitamin D deficiency and EMCS performed on maternal indication. A trend was observed towards higher odds of EMCS performed on maternal indication among vitamin D deficient women, but only among women who did not receive epidural anaesthesia (AOR = 0.285, p = 0.093).

**Table 3:** Associations between vitamin D deficiency and emergency caesarean section

	Without epidural anesthesia			With epidural anesthesia		
	OR	95% CI	P	OR	95% CI	P
Vitamin D status						
25OHD ≥ 30 nmol/L (ref)						
25OHD < 30 nmol/L	2.038	1.010–4.110	0.047	0.462	0.123–1.734	0.252
Obesity						
No (ref)						
Yes	0.817	0.366–1.823	0.621	0.675	0.316–1.441	0.309
Birth history						
No previous delivery (ref)						
Previous vaginal delivery	0.231	0.127–0.423	< 0.001	0.312	0.132–0.734	0.008
Previous delivery by cesarean	1.592	0.805–3.146	0.181	4.419	2.100–9.299	< 0.001
Season at sampling						
May-Oct (ref)						
Nov-Apr	0.853	0.514–1.416	0.538	1.801	1.020–3.179	0.042
Age (years)	1.080	1.023–1.141	0.006	1.072	1.008–1.139	0.026
Maternal height (cm)	0.960	0.925–0.997	0.033	0.942	0.901–0.985	0.009

*CI* confidence interval, *OR* odds ratio, *BMI* body mass index, *T3* third trimester, *25OHD* 25-hydroxyvitamin D

Vitamin D deficiency was related to significantly higher odds of birth asphyxia in multivariable logistic regression analysis, where deficient women had more than twice the odds (Table 4). Epidural anaesthesia did not modify the associations between vitamin D deficiency and birth asphyxia.

**Table 4:** Associations between vitamin D deficiency and birth asphyxia^a^

	OR	95% CI	P
Vitamin D status			
25OHD ≥ 30 nmol/L (ref)			
25OHD < 30 nmol/L	2.223	1.023–4.830	0.044
Obesity			
No (ref)			
Yes	1.758	0.861–3.590	0.122
Nulliparity			
No (ref)			
Yes	1.637	0.908–2.952	0.101
Season at sampling			
May- Oct (ref)			
Nov-Apr	0.532	0.310–0.914	0.022
Epidural anaesthesia at delivery			
No (ref)			
Yes	1.887	1.059–3.362	0.031
Maternal age (years)	1.034	0.978–1.094	0.242
Maternal height (cm)	0.964	0.925–1.004	0.080

*CI* confidence interval, *OR* odds ratio, *BMI* body mass index, *T3* third trimester, *25OHD* 25-hydroxyvitamin D

^a^Defined as either arterial umbilical cord pH < 7.1 or 5 min Apgar score < 7

Sensitivity analysis imputing missing data on vitamin D status did not alter the results on either birth asphyxia or EMCS (data not shown).

## Discussion

The results of this study show that vitamin D deficiency, defined as 25OHD < 30 nmol/L, in late pregnancy is associated with doubled odds of birth asphyxia, and EMCS in deliveries where epidural anaesthesia was not administered.

This study is one of the first to show that maternal vitamin D deficiency might be a risk factor for birth asphyxia as few studies have been conducted in the area. We found doubled odds of birth asphyxia and a higher prevalence of low Apgar score among women with vitamin D deficiency. One previous Swedish study has been conducted and found that children of women with 25OHD concentration < 50 nmol/L had approximately doubled incidence of birth asphyxia (Lindqvist et al. 2016), using a similar definition as in the current paper. However, not all studies have yielded the same results (Zhou et al. 2014) to which a lack of statistical power might have been a contributing factor. As birth asphyxia can have serious implications for the child, including increased mortality and neurological disabilities (Thornberg et al. 1995), the findings warrant further investigation of mechanisms and to verify causality.

Previous studies from America (Merewood et al. 2009; Scholl et al. 2012) link poor vitamin D status to C-section or EMCS. Studies from the UK (Savvidou et al. 2012) and China (Zhou et al. 2014; Yuan et al. 2017) found however no such association. Possible reasons why previous studies have yielded heterogeneous results include variation in C-section rate and disparate definitions of C-section. Some studies have included women who had undergone elective C-sections in their outcome, and these women could be at lower risk of vitamin D deficiency due to factors such as lower BMI, better health literacy, higher education level and increased likelihood of supplement use. The current finding that epidural anaesthesia modifies the association between vitamin D and EMCS has not been considered in previous studies, and is a possible explanation for the previous disparate results. Epidural anaesthesia at delivery is known to be associated with failure to progress (Cunningham et al. 2005), and is in itself a risk factor of EMCS. Possibly, this increased risk overshadowed any associations with vitamin D deficiency in deliveries where epidural was administered.

The main hypothesized biological mechanisms that link poor maternal vitamin D status with EMCS and birth asphyxia, is through interactions with the uterine smooth muscle (Ceglia et al. 2013). Poor vitamin D status is speculated to impair myometrial function due to either impaired regulation of intracellular calcium concentration (Al Otaibi 2014), reduced binding to the vitamin D receptor in the uterine endometrium and myometrium (Vienonen et al. 2004) or increased inflammation-induced cytokines and contractile associated factors in the myometrial smooth muscle cells (Thota et al. 2014). Our findings relating vitamin D deficiency to EMCS in the absence of epidural anaesthesia, in particular performed on maternal indication, provide some support for an effect of vitamin D on myometrial function. However, the underlying causes are unknown and requires further investigation.

Limitations of the current work include a potential lack of statistical power due to the relatively few cases of birth asphyxia and the low EMCS rate. Despite the cohort size of ~ 1800 women, birth asphyxia was only seen in 58 infants and EMCS in 141 women. Still, the statistically significant associations observed indicates that power was sufficient. Still, verification in larger cohorts may provide more robust effect estimates. Strengths of the current study include the prospective study design and the relativity large cohort size that enabled the investigation of vitamin D status and delivery outcomes. The population-based cohort also provided high external validity and generalisability of the results. An additional strength is the LC-MS/MS method for 25OHD analysis, which was performed by a laboratory included in the Vitamin D External Quality Assessment Scheme. Lastly, the use of medical chart data from pregnancy and delivery provided reliable outcome measures.

## Conclusions for Practice

In this Swedish prospective population-based cohort study, vitamin D deficiency in the third trimester of pregnancy was associated with a two-fold increased risk of birth asphyxia and EMCS in the absence of epidural anaesthesia. Prevention of vitamin D deficiency among pregnant women may reduce the incidence of birth asphyxia and EMCS. The mechanism behind the findings require further investigation.
